# Subpleural multilevel intercostal continuous analgesia after thoracoscopic pulmonary resection: a pilot study

**DOI:** 10.1186/s13019-019-1003-y

**Published:** 2019-10-22

**Authors:** Jelle E. Bousema, Esther M. Dias, Sander M. Hagen, Bastiaan Govaert, Patrick Meijer, Frank J. C. van den Broek

**Affiliations:** 1 Department of Surgery, Máxima Medical Centre, PO BOX 7777, Veldhoven, MB 5500 the Netherlands; 2 Department of Anaesthesiology, Máxima Medical Centre, Veldhoven, the Netherlands

**Keywords:** Thoracic surgery, Video-assisted thoracoscopic surgery, Pain management, Local anaesthetics

## Abstract

**Background:**

Sufficient pain control and rapid mobilisation after VATS are important to enhance recovery and prevent complications. Thoracic epidural analgesia (TEA) is the gold standard, but failure rates of 9–30% have been described. In addition, TEA reduces patient mobilisation and bladder function. Subpleural continuous analgesia (SCA) is a regional analgesic technique that is placed under direct thoracoscopic vision and is not associated with the mentioned disadvantages of TEA. The objective of this study was to assess surgical feasibility, pain control and patient satisfaction of SCA.

**Methods:**

Observational pilot study in patients who underwent VATS pulmonary resection and received SCA (*n* = 23). Pain scores (numeric rating scale 0–10) and patient satisfaction (5-point Likert scale) were collected on postoperative day (POD) 0–3. Secondary outcomes were the period of urinary catheter use and period to full mobilisation.

**Results:**

Placement of the subpleural catheter took an average of 11 min (SD 5) and was successful in all patients. Pain scores on POD 0–3 were 1.2 (SD 1.2), 2.0 (SD 1.9), 1.7 (SD 1.5) and 1.2 (SD 1.1) respectively. On POD 0–3 at least 79% of patients were satisfied or very satisfied on pain relief and mobilisation. The duration of subpleural continuous analgesia was 4 days (IQR 3–5, range 2–11). Urinary catheters were used zero days (IQR 0–1, range 0–6) and full mobilisation was achieved on POD 2 (IQR 1–2, range 1–6).

**Conclusion:**

Subpleural continuous analgesia in VATS pulmonary resection is feasible and provides adequate pain control and good patient satisfaction.

**Trial registration:**

This pilot study was not registered in a trial register.

## Introduction

Sufficient pain control and rapid mobilisation after video-assisted thoracoscopic surgery (VATS) are important to improve recovery and prevent postoperative pulmonary complications [1]. Thoracic epidural analgesia (TEA) is the gold standard for postoperative pain management following thoracic surgery [2]. When placed correctly the analgesic effect of TEA is clear, but failure rates of 9–30% have been described and awake placement can be stressful for patients [3–5]. In addition, when effective, TEA is associated with disadvantages such as immobilisation, bladder dysfunction and hypotension [6].

These disadvantages supported the development of other regional techniques for pain management. Single-shot thoracic paravertebral blockade, intercostal nerve blockade and serratus anterior blocks have been shown to be safe and effective for pain management after VATS [2, 7–9]. Recently several case-reports on successful single-shot and continuous infusion erector spinae block for postoperative analgesia after VATS have been reported [10–12]. A meta-analysis on single-injection versus continuous peripheral nerve blockade showed improved pain control, decreased need for opioids and greater patient satisfaction with the continuous infusion technique [13].

Subpleural continuous analgesia (SCA) is a regional multilevel intercostal analgesic technique to control postoperative pain using a subpleural catheter placed under general anaesthesia and direct thoracoscopic vision. It does not influence mobility, bladder function or blood pressure. Jung et al. retrospectively compared SCA with intravenous patient controlled analgesia for pain management after VATS pulmonary resection and found average pain scores (numeric intensity pain scale 0–10) higher than 3 on postoperative day (POD) 0–2 [14]. The effectiveness of SCA is hereby doubtful since Ried et al. presented pain scores (numeric analog scale 0–10) of 2.0 with TEA and 2.1 with single level intercostal continuous analgesia after thoracotomy for pulmonary resection [15]. The objective of this pilot study was to examine surgical feasibility, postoperative pain control and patient satisfaction of SCA in patients who underwent VATS pulmonary resection.

## Materials and methods

### Hypothesis

Subpleural continuous analgesia is feasible, provides adequate pain control and has good patient satisfaction in patients after VATS pulmonary resection.

### Study design and population

We performed an observational pilot study in a Dutch teaching hospital (Máxima MC, Veldhoven). 23 consecutive patients who underwent VATS pulmonary resection and had an indication for TEA (i.e. anatomical resection or major wedge resection) between April and September 2018 were included and received SCA for pain management instead of TEA. The pain scores and postoperative outcomes of SCA patients were compared to a historical group to benchmark our results. Since this was an observational pilot study we were not aiming to prove non-inferiority or even superiority of either technique. The historical group contained 23 consecutive patients who underwent VATS pulmonary resection and received TEA between April and September 2017.

### Primary and secondary outcomes

Primary outcomes were surgical feasibility, pain control and patient satisfaction on postoperative day (POD) 0–3. Secondary outcomes were length of hospital stay, chest tube drainage and urinary catheter use, incidence of postoperative hypotension and degree of postoperative mobilisation.

### Data collection

To assess surgical feasibility the duration of placement (minutes) and success of placement were collected. A numeric rating scale (NRS 0–10) was used to assess the level of pain in rest, while coughing and while moving. In addition, the necessity for intravenous and oral analgesics and the presence of postoperative nausea and vomiting were registered. Patient satisfaction on pain relief and mobilisation was measured on a 5-point Likert scale (very unsatisfied – unsatisfied – neutral – satisfied – very satisfied).

For secondary outcomes we collected length of postoperative hospital stay (days), length of chest tube drainage (days), the length of urinary catheter use (days) and the incidence of postoperative complications (hypotension, pneumonia, atelectasis, deep venous thrombosis, urinary tract infection, cardiovascular incidents and constipation). To examine hypotension we collected the lowest reported blood pressure in the morning, afternoon and evening on POD 0–3. Postoperative mobility was scored as the maximum achieved score on a non-validated 6-point scale (only in bed – sitting on the bedside – standing next to the bed – transfer from bed to chair – transfer from bed to bathroom – walking around the ward). Pain control, patient satisfaction and mobility scores were assessed on the recovery room (only pain scores), at the evening after surgery and in the morning, afternoon and evening on POD day 1 and 2 and in the morning and evening on POD 3. In order to describe the use of SCA we collected details on the SCA catheter placement (perforation of the parietal pleura (yes/no)) and possible complications in the postoperative period (leakage of fluid, SCA duration and ropivacaine-related adverse effects such as confusion, tingling sensation around the mouth, fasciculation, hallucination). Finally, we collected the following data from the electronical medical records: age, gender, length, weight, blood pressure at the pre-operative visit, American Society of Anaesthesiologists (ASA)-classification, indication for surgery (diagnostic, malignant lung or malignant metastatic), surgical details (single or multi-port VATS, duration, resected part of the lung, number of chest tubes).

In the historical TEA group prospectively collected pain scores on POD 1–3 were available. The baseline characteristics and postoperative outcomes data were collected retrospectively from the electronical medical records.

### Statistical analysis

Results were reported according to the Strengthening the Reporting of Observational studies in Epidemiology guidelines for observational studies [16]. The mean duration of placement of the catheter was calculated to describe surgical feasibility. Pain control was calculated as the mean pain scores in rest per day. To summarize the results and compare the groups we calculated mean pain scores on POD 1–3. We converted all opioids to an equivalent oral morphine dose in order to equally compare opioid use in both groups [17]. Patient satisfaction scores were reported as the proportion of patient who were ‘satisfied’ (score 4 out of 5) or ‘very satisfied’ (score 5 out of 5). The mean arterial blood pressure (MAP) was calculated by the following formula: ((diastole*2) + systole)/3. The presence of hypotension was defined as a systolic blood pressure below 90 mmHg or a greater than 30% decrease of the systolic blood pressure compared to the pre-operative visit. The ability of postoperative mobilisation was calculated as the day (median) that patients were able to walk to the bathroom and the day (median) that patients were able to walk around the ward.

Perforation of the parietal pleura during the placement of the subpleural catheter could have led to a decreased analgesic dose around the intercostal nerves due to leakage of analgesics into the pleural cavity. Therefore, we divided the SCA patients in a perforation and no perforation group and compared baseline characteristics, surgical details and pain scores between these groups.

Descriptive data were presented as means (with standard deviation (SD) and/or range) or medians (with interquartile range (IQR) and/or range) depending on (normally or skewed) distribution of data. Categorical data were presented as counts and percentages. Categorical data were compared by the Chi-square test, whereas numerical data were compared by the unpaired T-test or Mann-Whitney U test depending on (normally or skewed) distribution of data. We calculated 95% confidence intervals (95%-CI) around proportions using the Wilson interval [18]. All performed tests were two-sided and we set the significance threshold at *p* = .05. All calculations and statistical analysis were done using the Statistical Package for Social Sciences, version 22.0 (SPSS Inc., Chicago, IL, USA).

### General anaesthesia and postoperative additional analgesia

All patients received general anaesthesia using propofol, sufentanil and rocuronium for induction and either propofol or sevoflurane for maintenance of anaesthesia (at the discretion of the anaesthesiologist). After surgery patients received additional analgesics according to the analgesic pain relief ladder; oral paracetamol (standard dose of 4000 mg per 24 h) with additional nonsteroidal anti-inflammatory drugs (NSAIDs, Naproxen, when patients had no contra-indication 1000 mg per 24 h) and if necessary morphine or morphine equivalent (oral or intravenous).

### Subpleural pain catheter placement and protocol

**Fig. 1:**
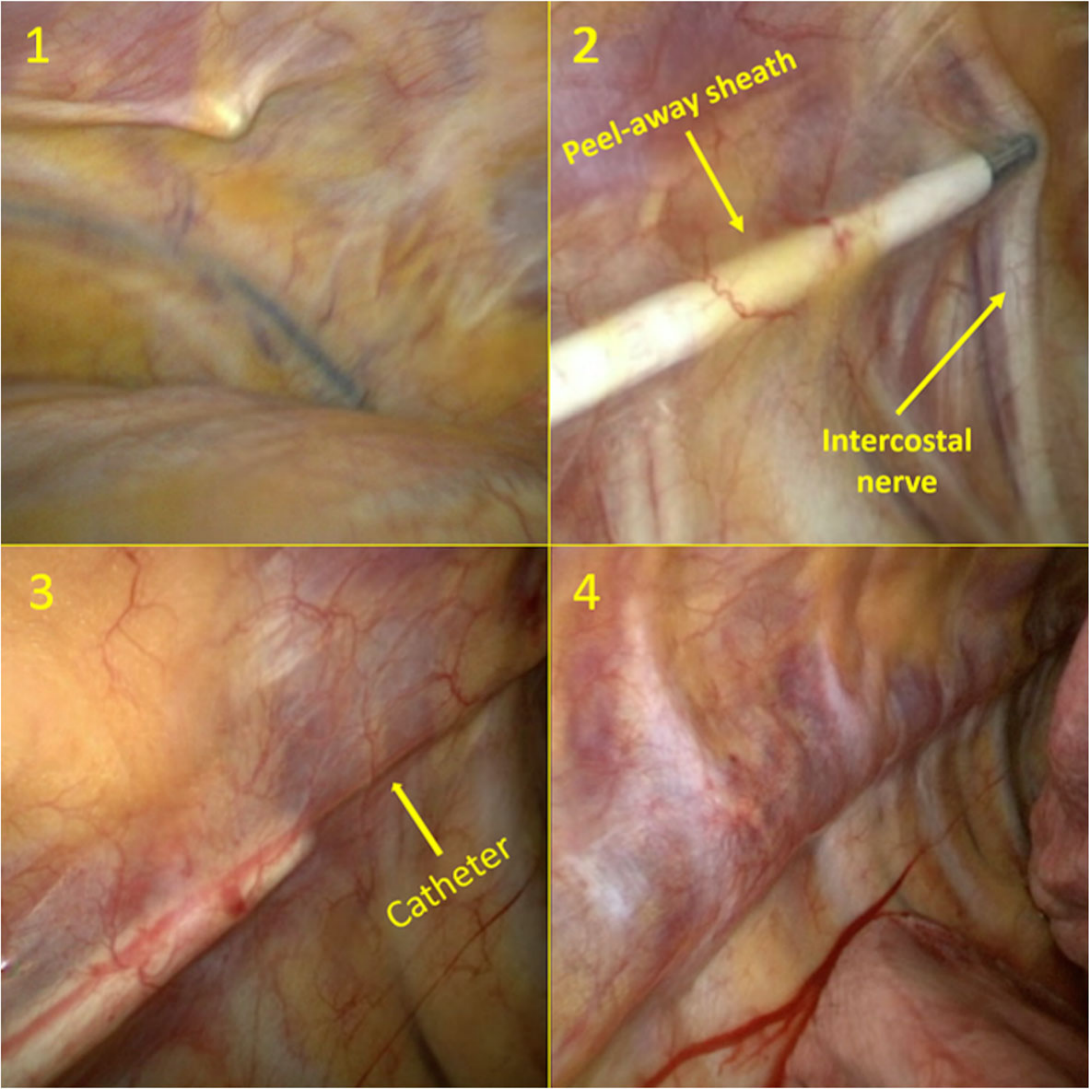
Subpleural catheter placement. Subpleural catheter placement. 1: introduction of the tunnelling device in the subpleural space; 2: moving the tunnelling device upwards; 3: removing the peel-away sheath; 4: multilevel subpleural catheter with 40 ml ropivacaine 2 mg/ml bolus

The multi-orifice 25 cm length On-Q local anaesthetic infiltrating catheter (On-Q: Avanos, Alpharetta, GA) was placed intraoperatively under direct thoracoscopic vision by one of two lung surgeons (FvdB, BG). After general anaesthesia and endotracheal intubation the patient was positioned in the lateral decubitus position with the affected side up. Access to perform was achieved by either uni- or multiportal incision(s). The pain catheter was inserted dorsally and at least two costal levels caudally of the most caudal placed incision which is generally made in the 8th intercostal space. After skin incision we searched for the subpleural space using blunt dissection under direct thoracoscopic vision (Fig. 1, image 1). The complete tunnelling device was moved cephalad using hydro-dissection (using sterile saline solution 0.9%) by which method the level above the most cephalad incision was always reached. The catheter was placed approximately 5 cm laterally from the vertebra and the thoracic sympathetic chain covering the intercostal nerves on multiple levels (Fig. 1, image 2). After adequate placement of the tunnelling device the stylet is retracted, the infiltrating catheter is inserted and then the peel away sheath is removed (Fig. 1, image 3). Finally, we created an anterior subcutaneous tunnel of approximately 10–15 cm to prevent leakage of local anaesthetic through the skin incision and have a more convenient (anterior) insertion point for the patient. All patients received a direct 40 ml (ml) bolus ropivacaine 2 mg/ml (Fig. 1, image 4). According to our local hospital protocol the continuous ropivacaine 2 mg/ml infusion was started after bolus in the operating room or on the recovery room with an infusion rate of 10 ml/hour.

## Results

A total of 23 patients (mean age 68 years (SD 6), 74% males) were included for analysis. The primary surgical technique was either uniportal (*n* = 4) or conventional (*n* = 19) VATS. 18 Patients (78%) underwent an anatomical resection (i.e. segmentectomy, lobectomy or bilobectomy) and in the remaining five patients a large wedge was resected. We did a lobe-specific mediastinal lymph node dissection in 16 patients (70%). All patients received one chest tube for postoperative drainage (Table 1).

**Table 1:** Clinical characteristics of patients in the study

	SCA patients (*n* = 23)	TEA patients (*n* = 23)	*P* Value
Age, mean (SD), y	68 (6)	63 (12)	.07
Sex, No. (%)			
Male	17 (74)	8 (35)	.01
Female	6 (26)	15 (65)	
ASA-classification, No. (%)			
ASA-1	3 (13)	0	.19
ASA-2	14 (61)	17 (74)	
ASA-3	6 (26)	6 (26)	
Indication for surgery, No. (%)			
Diagnostic	1 (4)	3 (13)	.36
Malignant – Lung	16 (70)	17 (74)	
Malignant – Metastasis	6 (26)	3 (13)	
Tumour localization, No. (%)			
Right upper lobe	9 (40)	7 (30)	.83
Right middle lobe	3 (13)	2 (9)	
Right lower lobe	3 (13)	5 (22)	
Left upper lobe	4 (17)	6 (26)	
Left lower lobe	4 (17)	3 (13)	
Surgical technique, No. (%)			
VATS single-port	4 (17)	6 (26)	.48
VATS multi-port	19 (83)	17 (74)	
Resection, No. (%)			
Wedge	5 (22)	7 (30)	.51
Segmentectomy	1 (4)	0	
Lobectomy	16 (70)	16 (70)	
Bilobectomy	1 (4)	0	
Lymph node dissection, No. (%)			
None	5 (22)	6 (26)	.81
Ipsilateral hilair	2 (8)	3 (13)	
Ipsilateral hilair + mediastinal	16 (70)	14 (61)	
Surgery duration, mean (SD), min	153 (52)	130 (74)	.23

*SCA* subpleural continuous analgesia, *TEA* thoracic epidural analgesia, *SD* standard deviation, *y* years, *No.* number, *ASA* American Society of Anaesthesiologists, *VATS* video-assisted thoracoscopic surgery, *min* minutes

### Primary outcomes

Placement of the subpleural catheter took on average 11 min (SD 5, range 4–24) and was successful in all patients. The mean pain score in rest on the recovery room was 1.1 (SD 1.2) and on the evening after surgery it was 3.1 (SD 2.0). On POD 1–3 the mean pain scores in rest were 2.0 (SD 1.9), 1.7 (SD 1.5) and 1.2 (SD 1.1) respectively. The mean pain score in rest on POD 1–3 together was 1.6 (SD 1.3) (Table 2). Mean pain scores while moving were 4.2 (SD 1.9), 3.6 (SD 2.5), 3.1 (SD 1.9) and 3.1 (SD 2.3) on POD 0–3 respectively. Mean pain scores while coughing were 4.4 (SD 2.1), 4.0 (SD 2.1), 3.3 (SD 2.2) and 3.2 (SD 2.1) on POD 0–3 respectively. Patient controlled intravenous morphine was used in 15 patients (65%), the remaining 8 patient received oral morphine on request. The total morphine consumption was 23.4 mg (SD 30.0), 17.0 mg (SD 28.4) and 6.9 mg (SD 12.7) on POD 1–3 respectively (Table 2). With SCA, 79% (15/19; 95%-CI 57–92) of patients were satisfied or very satisfied on the evening after surgery (due to prolonged surgery and anaesthesia satisfaction scores on the evening after surgery were missing in 4 patients), whereas 78% (18/23; 95%-CI 58–90), 83% (19/23; 95%-CI 63–93) and 90% (18/20;95%-CI 69–97) of patients were satisfied or very satisfied on POD 1–3 respectively.

**Table 2:** Postoperative pain scores in rest, mean (SD) and additional opioid use (mg), mean (SD)

	SCA (*n* = 23)	TEA (*n* = 23)	*P* value
Recovery room	1.2 (1.2)	0.6 (0.9)	.43
Postoperative day 1			
Morning	2.4 (2.0)	2.3 (2.3)	.91
Noon	2.2 (1.8)	1.5 (1.8)	.27
Evening	1.6 (1.9)	1.7 (2.2)	.91
Mean	2.0 (1.9)	1.9 (1.6)	.77
Additional opioids*	23.4 (30.0)	3.9 (10.5)	<.01
Postoperative day 2			
Morning	2.2 (1.9)	2.0 (2.2)	.79
Noon	1.6 (1.6)	1.9 (1.9)	.55
Evening	1.7 (1.7)	2.4 (2.1)	.32
Mean	1.7 (1.5)	1.9 (1.6)	.65
Additional opioids*	17.0 (28.4)	4.3 (8.2)	.05
Postoperative day 3			
Morning	1.4 (1.2)	1.5 (1.9)	.73
Evening	1.1 (1.1)	1.6 (1.9)	.44
Mean	1.2 (1.1)	1.6 (1.2)	.39
Additional opioids*	6.9 (12.7)	5.0 (8.7)	.60
Postoperative day 1–3			
Mean	1.6 (1.3)	1.7 (1.2)	.78

*SCA* subpleural continuous analgesia, *TEA* thoracic epidural analgesia, *SD* standard deviation. *mg* milligram, *POD* postoperative day, *Sum of intravenous morphine by patient controlled analgesia and oral Oxycodone. For this sum intravenous morphine dosage is doubled based on the Opioid Conversion Chart

### Secondary outcomes

The length of postoperative hospital stay was 4 days (IQR 3–8, range 2–18) with a period of chest tube drainage of 2 days (IQR 1–4, range 1–13). The period of urinary catheter use was 0 days (IQR 0–1, range 0–6). At the pre-operative visit none of the patients suffered from hypotension with a mean MAP of 100 mmHg (SD 12). Hypotension during the first three postoperative days was seen in only one patient (5%). SCA patients were able to walk to the bathroom without help on median POD 1 (IQR 1–2, range 1–6). Walking around the ward without help was median possible on POD 2 (IQR 1–2, range 1–6). Postoperative complications occurred in seven patients (30%) (Table 3).

**Table 3:** Postoperative complications

TEA (*n* = 23)	
2	Pneumonia treated with antibiotics
1	Subcutaneous emphysema (no intervention)
1	Delirium requiring medication
1	Ulnar neuropathy
1	Atelectasis requiring bronchoscopy
SCA (*n* = 23**)**	
1	Pneumonia treated with antibiotics
1	Subcutaneous emphysema requiring extra chest drain
1	Laryngeal nerve palsy
1	Ischemic stroke
1	Prolonged air leak (no intervention)
1	Prolonged air leak requiring surgical treatment
1	Constipation treated with laxatives

*TEA* thoracic epidural analgesia, *SCA* subpleural continuous analgesia

### Subpleural continuous analgesia

The duration of subpleural continuous analgesia was 4 days (IQR 3–5, range 2–11). One patient reported deafness of the skin around the location where the catheter was inserted. Leakage of local anaesthetic from the skin insertion place of the pain catheter occurred in 6 patients (23%). We saw no other SCA-catheter related complications or ropivacaine related systemic side effects. Minor perforation of the parietal pleura during placement of the catheter occurred in 12 patients (52%). None of these patient had abundant drain production and no additional interventions were required to repair the perforations. We found no significant differences between the no pleural perforation group (*n* = 11) and the pleural perforation group (*n* = 12) in left or right sided surgery, duration of the placement of the pain catheter, mobilisation and pain scores in rest, while coughing or while moving on POD 0–3.

### Historical group: patients with TEA

Baseline and surgical characteristics of the historical group were similar to the SCA patients, except for gender (more females in the TEA group; *p* = 0.01) (Table 1). The mean pain score in rest on POD 1–3 in TEA patients was 1.7 (SD 1.2), which was similar to the mean pain score in SCA patients on POD 1–3 (1.6 (SD 1.3); *p* = .78) (Table 2). Significant lower mean doses of morphine were used in TEA patients on the first two postoperative days, no significant differences were found on day three (Table 2). Hypotension on POD 1–3 was found in 26% (6/23) of TEA patients versus 5% (1/23) of SCA patients (*p* = .04), while the pre-operative MAP was comparable between groups (TEA 95 mmHg vs SCA 110 mmHg, *p* = .20). The length of hospital stay (4 days, IQR 3–5) and chest tube drainage (2 days, IQR 1–3) were similar to SCA patients. The period of urinary catheter use was 2 days (IQR 2–3, range 0–5) which was significantly longer compared to patients with SCA (0 days, IQR 0–1; *p* < .01). Postoperative complications occurred in seven patients (30%) with SCA and six patients (26%) with TEA (*p* = .74) (Table 3).

## Discussion

Sufficient pain control after VATS enables adequate breathing and early mobilisation hereby reducing postoperative complications and enhance recovery. Our results suggest that subpleural continuous analgesia in VATS pulmonary resection is feasible and provides adequate pain management and good patient satisfaction.

To the best of our knowledge no consensus on optimal postoperative pain management after VATS is achieved in current literature. A review of enhanced recovery after thoracic surgery (ERATS) protocols strengthened the lack of unambiguity. The five included protocols all used different techniques for postoperative pain management: oral, intravenous, intercostal, paravertebral and epidural anaesthesia [19]. The use of continuous analgesia in the subpleural space is previously described after thoracic surgery. Hotta et al. provided a randomized trial on extrapleural continuous analgesia versus TEA in VATS patients in 2011. They found no significant differences in visual analog scale pain scores and the need for rescue analgesia [20]. Jung et al. compared SCA to intravenous patient controlled analgesia and found comparable average pain scores [14]. Surgical feasibility, mobilisation and patient satisfaction were however not included in these studies. Patient comfort and satisfaction are important factors for enhanced recovery. Patient comfort appears to be a complex combination of multiple elements, while pain and mobility are measurable elements of it [21]. One of the most important advantages of local analgesia is the ability of early mobilisation. Patients with adequately placed TEA are often judged as ‘immobilized’ despite intact motor function of the legs. According to our local protocol TEA patients are allowed to mobilize under supervision (doctor, nurse or physiotherapist) when the motor function and sensibility are intact. In daily practice intensive supervision will not be available, resulting in immobilization during TEA. Since SCA is not associated with loss of motor function patients are allowed to mobilize independently immediately after surgery. This resulted in earlier mobilisation in SCA patients compared to TEA patients.

Urinary catheters could be another cause of restricted postoperative mobilisation. Its use combined with TEA is however common practice in most hospitals. A prospective study showed significant post-void residuals in patients with thoracic epidural analgesia [22]. In a randomized controlled trial significant more urinary problems (urinary catheter reinsertion, higher rate of bladder scans and more in-and-out catheterization) were found in patients with early urinary catheter removal (48 h postoperative) compared to patients whose urinary catheter is removed six hours after terminating TEA [23]. Therefore, urinary catheters appear mandatory in TEA while in SCA patients bladder function remains normal.

Sufficient pain management allows patients to mobilize. Insufficient pain control by epidural or local analgesia could be compensated by additional oral or intravenous analgesics. However, since opioids and NSAIDs have certain disadvantages it is important to minimalize the use of these drugs. SCA patients had a higher opioid consumption on POD 1 and 2. These results are in concordance with current literature where slightly higher additional analgesic use is described in continuous local analgesia groups versus TEA-groups in thoracic surgery [15, 24]. However, the mean total opioid dose on the first and second POD (23.4 mg and 17.0 mg respectively) was in our opinion acceptable. The mean dose on POD 3 already decreased to 6.9 mg respectively and we saw only one patient with constipation which was successfully treated with laxatives.

Placement of the SCA catheter was easy and safe. Placement of the catheter took an average of 11 min resulting in a non-significant prolonged operation time in SCA patients. We noticed a very steep learning curve since the placement of the first 12 catheters took an average of 14 min versus 8 min in the last 11 catheters.

The main benefits of SCA compared to TEA and other regional analgesic techniques (paravertebral block, intercostal block, erector spinae block) were fast placement under direct thoracoscopic vision and no need of additional equipment or specialized staff (beside the catheter). In addition, patients benefit from the placement of the analgesic catheter under general anaesthesia compared to possible stressful awake placement of a thoracic epidural catheter or awake percutaneous regional analgesic techniques [25]. Lastly, SCA provides continued instead of single shot analgesia and therefore has longer duration of pain control.

The main limitations of our study were the observational character and the small sample size. This may have led to an underestimation of differences among TEA and SCA due to insufficient power. Since the primary objective was feasibility, pain control and patients satisfaction of SCA the results of the comparison with the historical group should be interpreted with care.

The results and limitations of our study and the lack of consensus in current literature strengthen the need for prospective, randomised research on the most (cost-)effective technique of postoperative pain management after VATS pulmonary resection. Results of future research may aide in optimizing patient care and improve ERATS protocols.

## Conclusion

Subpleural continuous analgesia in VATS pulmonary resection is feasible and provides adequate pain control and good patient satisfaction.

## Data Availability

The dataset used during the current study is available from the corresponding author on reasonable request.

## Abbreviations

95%-CI: 95% confidence interval
ASA: American society of anaesthesiologists
ERATS: Enhanced recovery after thoracic surgery
IQR: Interquartile range
MAP: Mean arterial pressure
mg: milligram
min: minutes
ml: millilitre
No.: Number
NRS: Numeric rating scale
NSAIDs: Nonsteroidal anti-inflammatory drugs
POD: Postoperative day
SCA: Subpleural continuous analgesia
SD: Standard deviation
TEA: Thoracic epidural analgesia;
VATS: Video-assisted thoracoscopic surgery
y: years
